# Divergent proteomic profiles of opium poppy cultivars

**DOI:** 10.55730/1300-0152.2684

**Published:** 2024-02-06

**Authors:** Setenay AYKANAT, Mine TÜRKTAŞ

**Affiliations:** Department of Biology, Faculty of Science, Gazi University, Ankara, Turkiye

**Keywords:** Benzylisoquinoline alkaloid (BIA), bioinformatics, LC/MS-MS, opium poppy, plant proteomics

## Abstract

We examined the proteomic profiles of three registered opium poppy cultivars (*Papaver somniferum* L.) with varying alkaloid contents. The study was conducted on both the stem and capsule organs. A high number of differentially expressed proteins (DEPs) were identified between the cultivars and the organs. We analyzed DEPs for their contribution in GO terms and KEGG pathways. The upregulated DEPs were significantly enriched in photosynthesis and translation for morphine-rich and noscapine-rich cultivars, respectively. The data indicated that photosynthesis is crucial for benzylisoquinoline alkaloid (BIA) biosynthesis, but different processes are also effective in morphine and noscapine biosynthesis, which occur at different branches in the biosynthetic pathway. The proteomics profiles revealed that energy demand is more effective in morphine biosynthesis, while translational control plays a leading role in noscapine biosynthesis. This study represents the first report demonstrating organ-based and cultivar-based protein expression differences in mature poppy plants.

## 1.Introduction

Opium poppy (*Papaver somniferum* L.), a member of the Papaveraceae family of the Papaverales order, is a medicinal and industrial plant species with many different uses and significant economic value. The primary significance of the opium poppy arises from its the benzylisoquinoline alkaloids (BIA), which are structurally different group of secondary metabolites specific to the plants it produces. Although alkaloids were found in approximately 20% of plants, the opium poppy is the sole producer of morphinan alkaloids. Opium poppy produces many important BIAs, such as morphine, codeine, which exhibit narcotic analgesic effects; thebaine, which is used as a pain reliever; sanguinarine, which has antimicrobial properties; and noscapine, which has anticancer and antiviral properties (Singh et al., 2019). For these reasons, opium poppy is accepted as one of the most important natural product sources in the pharmaceutical industry (Bulut et al., 2020). To date, about 2500 metabolites belonging to the BIA group have been identified, and more than 80 alkaloids have been isolated from the opium poppy plant, most of which are intermediates in the complex multibranched BIA pathway (Zulak et al., 2007).

BIA biosynthesis is localized to sieve elements in the laticifer, while the opium alkaloids are mainly accumulated in the capsule (Bird et al., 2003; Onoyovwe et al., 2013). The BIA biosynthesis is a complex pathway, involving several enzymes (Singh et al., 2019). BIA alkaloids are a structurally differentiated group of tyrosine-derived metabolites (Li et al., 2020). BIA biosynthesis begins with the conversion of dopamine and 4-hydroxyphenylacetaldehyde (4-HPAA) derived from the amino acid tyrosine to (S)-norcoclaurine by the enzyme norcoclaurine synthase (NCS). Subsequently, (S)-norcoclaurine is converted to (S)-reticulin which is the central intermediate molecule. The last common intermediate molecule, (S)-reticulin, splits into different branches and terminates with either sanguinarine, noscapine, berberine or morphine alkaloids (Gurkok et al., 2015). Morphine, noscapine, thebaine, codeine, and papaverine are the major alkaloids, and morphine is the most abundant BIA produced in opium poppy (Zulak et al., 2007). While various alkaloids are synthesized by Papaver species, morphine is produced only by *P. somniferum* and *P. setgerum* species. On the other hand, Noscapine is among the major alkaloids in latex, exhibiting anticancer and antiviral properties alongside its utilization as a cough suppressant (Chen et al., 2015). Noscapine has also been shown to play a neuroprotective role in neurodegenerative diseases (Tomar et al., 2017). Moreover, interest in this alkaloid has further increased, given its nonaddictive and its recent identification as a drug candidate for treating the Covid-19 epidemic (Ebrahimi 2020; Kumar et al., 2022).

The amount and content of alkaloids vary depending on the region where the plant is grown, the conditions of obtaining them, and the climate, while the content also differs based on organs (Facchini and De Luca, 1995). The researchers improved the cultivars of opium poppy to obtain optimal opiate production through extensive breeding cycles. The protein content of an organism is much greater than its genome or transcriptome content. Considering that more than 80,000 protein-coding genes exist in the poppy genome (Guo et al., 2018), the diversity of proteins among cultivar is evident.

Many studies have been carried out to elucidate the pathways of secondary metabolites produced by the poppy plant. In this context, there are many transcriptome studies with different varieties of poppy (Pathak et al., 2013; Gurkok et al., 2015; Zhao et al., 2019; Subramaniyam et al., 2020; Pei et al., 2021). On the other hand, despite the large number of transcriptome studies in this field, there are significantly fewer proteomic studies. In the literature, there is limited information about the protein profiles of opium poppy cultivars. In a previous study, poppy latex was analyzed using two-dimensional gel electrophoresis, and 69 proteins were determined (Decker et al., 2000). In another study, proteins from cell suspension culture of *P. somniferum* cv Marianne treated with fungal elicitor were analyzed, and 219 proteins were determined (Zulak et al., 2009). While a linear relationship was observed between the amount of elicitor-induced transcripts and the amount of protein in the study, this correlation was not directly observed between the repressed transcripts. These results point to the extent to which posttranscriptional regulation is effective on genes in the BIA pathway in poppy. In another study conducted by the same researchers in 2010, proteome and transcriptome analyses were performed using cell suspension culture of *P. somniferum* cv Marianne cultivar, which were also treated with a fungal elicitor (Desgagne Penix et al., 2010). In that study, the alkaloid metabolism induced by fungal elicitor stimuli was specifically investigated for sanguinarine alkaloid. Although these studies provide essential data for the poppy plant, the metabolic profile of cell suspensions may differ from that of plant tissues, as these pathways are quite complex (Martinez Esteso et al., 2015). As stated in related studies, alkaloid accumulation does not occur continuously in poppy cell cultures. However, alkaloid biosynthesis takes place constantly in the poppy plant. Therefore, those studies do not provide holistic data on the entire protein content of plant organs, where the biosynthesis and accumulation of BIA alkaloids occur. In another study, latex and stem proteins isolated from the immature capsule of *P. somniferum* cv Roxanne cultivar were separated using SDS-PAGE technique and identified through mass spectrometry method (Onoyovwe et al., 2013). In a recent study, the effects of drought stress on newly germinated poppy plants were investigated in terms of proteomics (Kundratova et al., 2021). The study aims to determine the proteins in the newly developed poppy plants under drought stress, though no findings related to the BIA pathway were reported.

The abovementioned proteome studies were designed on poppy cell cultures which were induced by certain alkaloids with certain stimuli. However, as it is known, proteins interact with each other in an organism. Thus, the protein content in cell culture, where only certain alkaloids are induced by stimuli, is expected to differ from the protein profile in a mature poppy plant with a whole alkaloid content. Therefore, it is vital to analyze the steady-state protein profiles of mature poppy plants.

Türkiye is one of the few poppy producers in the world. Through extensive breeding studies conducted over many years by the sole authorized institution, Turkish Grain Board (TMO; Ankara, Türkiye), poppy varieties with high BIA contents have been developed and registered. (http://www.tmo.gov.tr/Upload/Document/Hashasileilgiligenelbilgiler.pdf). However, only a few molecular studies have been conducted on these varieties (Gurkok et al., 2015; Bulut et al., 2020). The study is also unique in its contribution to the evaluation and development of Turkish poppy cultivars.

Liquid chromatography-mass spectrometry/mass spectrometry (LC-MS/MS) is one of the most sensitive methods preferred for the determination of low amounts of analytes in the sample, as well as for the determination of multiple analytes. The method has a wide range of uses as it provides information about the amount, structure, and molecular weight of the components in the sample. Therefore, it is one of the most preferred methods in proteomic analyses.

In this study, the organ-based and cultivar-based protein profiles of mature poppy plants were comprehensively analyzed for the first time. In opium poppy, the bifurcation in the biosynthetic pathway of morphine and noscapine has not been elucidated. By analyzing both the control group and varieties rich in morphine and noscapine alkaloids together, the effects of proteins in this distinction were directly investigated for the first time. The data contribute to broadening our understanding of complex branches of the BIA metabolic network in opium poppy. In this way, the data is also of great importance in terms of initiating future studies, such as developing new varieties with high alkaloid content.

## 2. Materials and methods

### 2.1. Plant material

The three registered *Papaver somniferum* varieties developed by Turkish Grain Board (TMO) were used. The varieties differ in their benzylisoquinoline alkaloid (BIA) content. *P. somniferum* cv Ofis_96, with 0.6% morphine and 0.02% noscapine content, was used as a control, *P. somniferum* cv Ofis_1, with 1.8% morphine content, was used as a morphine-rich sample, and *P. somniferum* cv Ofis_NP, with 1.3% noscapine content, was used as a noscapine-rich sample. Samples were obtained from the cultivation field of the Afyon Alkaloids Plant Directorate in Afyonkarahisar Province, Bolvadin, in June. Samples were grown until seed formation, and young capsules and stems (3 cm in length) were collected in the blossom formation period, which is the most intense period of alkaloid production (Kim et al., 2018). The samples were immediately frozen in liquid nitrogen and stored at −80 °C until use.

### 2.2. Protein extraction and LC/MS-MS analysis

Three biological replicates were used for each opium poppy sample. The total protein from the samples was extracted using the Plant Protein Extraction Kit (Solarbio, Beijing, China), following the manufacturer’s instructions. The isolated protein samples were stored at −20 °C until use. The isolates were directly used for filter-aided sample preparation (FASP). The resulting peptides were analyzed by liquid chromatography–tandem mass spectrometry (LC-MS/MS). Xevo-G2-Si-QTOF was used for the LC-MS/MS analysis, and data was obtained with SONAR method. Mass range of m/z 50–2000 was used for the collection of the peptides. For the identification of proteins, 48,439 peptides containing the keyword “Papaver” were searched in the UniProt database (accessed 12.10.2022). Only peptides and proteins with a false discovery rate threshold under 0.01 were considered.

The data of this study is available with the accession numbers PXD046151 for ProteomeXchange and JPST002348 for jPOST.

### 2.3. Data analysis

The quality and quantity analyses of the proteins were performed using Progenesis QI software. A protein false discovery rate (FDR) of 1% was employed for evaluating peptide accuracy. The normalized protein abundances of three biological replicates of the samples were used to identify differential expressions between the samples. Normalization of protein abundances was performed using Progenesis QI software. The proteins meeting the criteria of p ≤ 0.05 and fold change (FC) ≥ 1, based on log_2_ expression value, were assigned as differentially expressed proteins (DEPs). Gene Ontology (GO) analysis was used to identify the identified proteins (Ashburner et al., 2000). During this process, three subontologies were examined: biological process (BS), cellular component (HB), and molecular function (MF). Ontology analyses were performed using the DAVID database (Dennis et al., 2003). Pathway analyses of the identified proteins were obtained from the KEGG database (Kanehisa and Goto 2000). Base R generated heatmap was used to visualize the hierarchical clustering of protein expression data. Protein-protein interaction networks of the resultant proteins were constructed using String (Szklarczyk et al., 2019) with an interaction score of ≥ 0.4.

## 3. Results

The LC-MS/MS analysis identified a total of 1606 proteins. Among them, 1202 proteins exhibited differential expression in any of the pairwise comparisons. Each cultivar shows a distinctive expression profile. To gain a better understanding of the protein profiles, the samples were compared based on the organs and cultivars.

### 3.1. Differentially expressed proteins-based on organ

**Figure 1:**
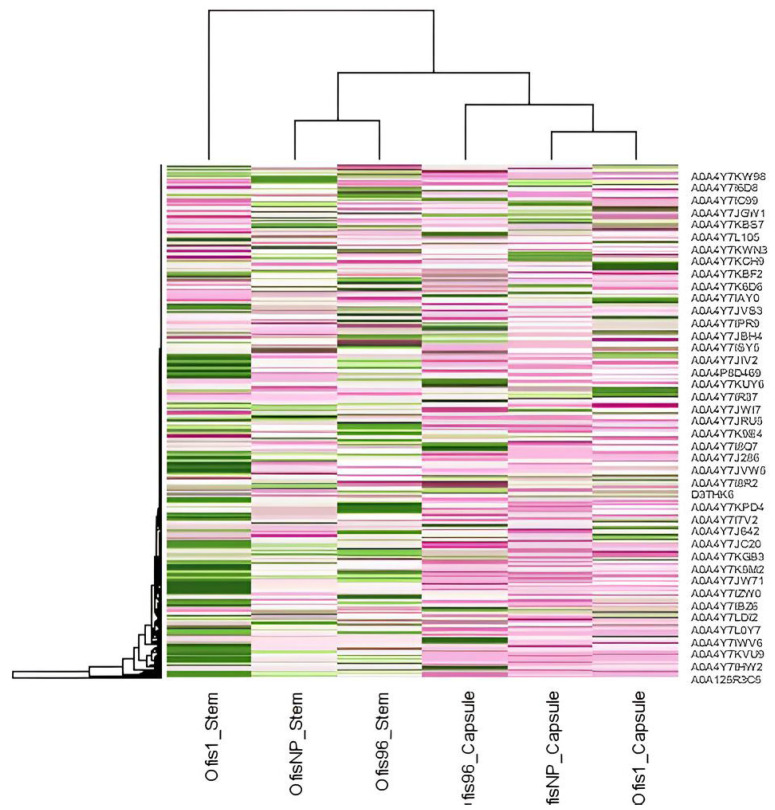
Heatmap displaying protein abundances of each sample. Colors show an increase of expression level from pink to green.

The stem and capsule proteins of each cultivar were compared to each other. We observed that an organ-based separation was evident, as indicated by the heatmap (Figure 1). The stems, where BIA biosynthesis takes place, and the capsules, where accumulation occurs, were clustered into two groups.

**Figure 2:**
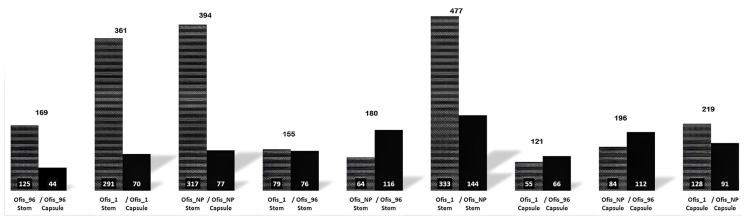
Number of DEPs between a) stem and capsule, b) cultivars. The total number of DEPs were given on each column. The number of upregulated proteins of the sample was given in each column.

**Figure 3:**
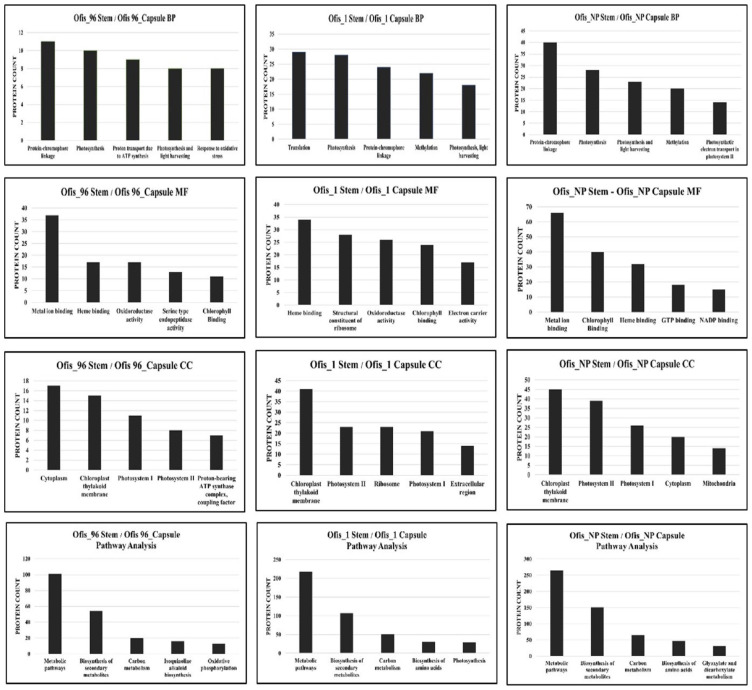
Top five enriched BP/MF/CC GO terms and KEGG pathways of DEPs between the organs.

When the protein differences in the organs were examined, it was observed that the majority of the proteins exhibited upregulation in stems compared to those in capsules in all three cultivars (Figure 2). The highest number of DEPs was found among the organs of alkaloid-rich cultivars, while the least number of DEPs was observed between the organs of Ofis_96. DEPs identified between stem and capsule were analyzed using GO term enrichment to investigate their functions. The proteins were classified based on molecular function, biological process, and cellular compartment ontology terms (Figure 3).

The analysis contains several Biological Process (BP) ontology terms and reveals a high proportion of proteins associated with “protein-chromophore linkage” and “photosynthesis” for all three cultivars. Additionally, “methylation” was one of the most abundant BP terms for the DEPs between the organs of alkaloid-rich cultivars. Many proteins were also assigned to GO Molecular Function (MF) and Cellular Compartment (CC) terms. A high prevalence of proteins exhibiting MF terms such as “metal ion binding” and “heme binding”, as well as CC terms such as “cytoplasm” and “chloroplast thylakoid membrane” were found.

In detail, proteins, especially those whose expression increased in stems relative to the capsules, are associated with BP term “photosynthesis” (Supplementary Figure 1). The number of these proteins in the organs of alkaloid-rich cultivars is even higher than in the organs of control cultivar. On the other hand, the proteins with increased expression in the capsules of alkaloid-rich cultivars were associated with “translation”, while the upregulated proteins in the capsule of Ofis_96 were involved in “fatty acid biosynthetic process” and “nutrient reservoir activity”.

The results of KEGG pathway analysis revealed that DEPs between the organs were mainly enriched in the biosynthesis of secondary metabolites and carbon metabolism pathways (Figure 3).

### 3.2. Differentially expressed proteins-based on cultivar

To analyze the protein differences between the three poppy varieties, the protein profiles were compared, and the number of DEPs is shown in Figure 2. The least number of differences were observed in the comparison between Ofis_1 capsule and Ofis_96 capsule. The number of proteins that differed the most was found between Ofis_1 stem and Ofis_NP stem (477 DEPs). The highest number of DEPs were observed in comparisons of Ofis_NP with the other two cultivars. Notably, the protein expressions were decreased in Ofis_NP compared to the those in both Ofis_1 and Ofis_96, and this profile is observed for both stem and capsule.

**Figure 4:**
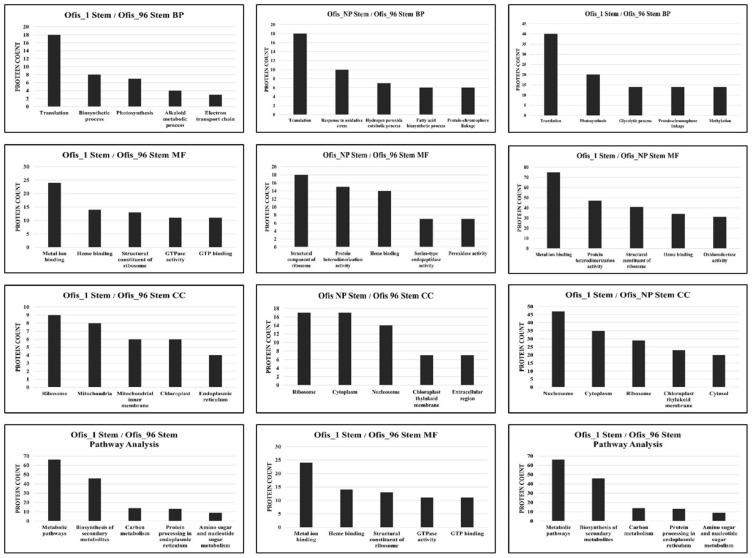
Top five enriched BP/MF/CC GO terms and KEGG pathways of DEPs between the stems of different cultivars.

The ontology analyses of the DEPs obtained from the stems of different cultivars revealed that “translation” was the most abundant BP term (Figure 4). Confirming that result, “ribosome”, where translation occurs, was found to be the most enriched CC term. In depth analysis of the DEPs obtained from comparison between the stems of the noscapine- and morphine-rich cultivars showed that the proteins involved mostly in “translation” were more expressed in noscapine-rich cultivar. Conversely, the proteins primarily associated with “photosynthesis” exhibited higher expression morphine-rich cultivar (Supplementary Figure 2).

KEGG analysis indicated that the largest number of DEPs between the stems of the cultivars were involved in the biosynthesis of secondary metabolites and carbon metabolism (Figure 4).

**Figure 5:**
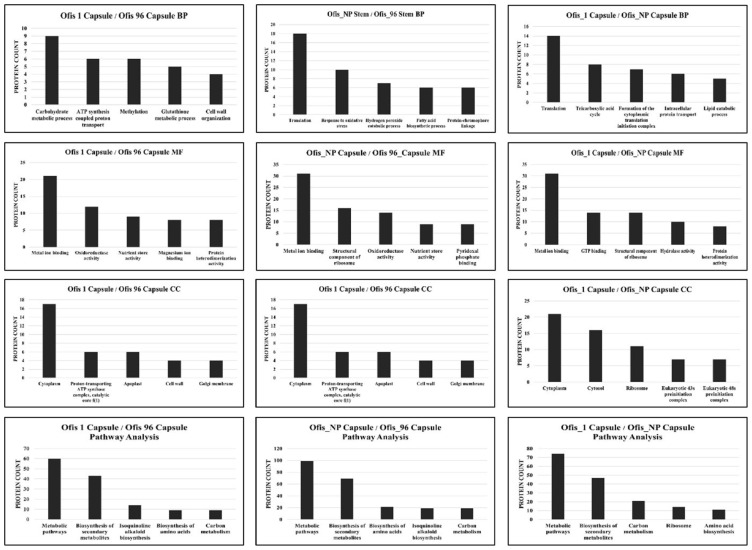
Top five enriched BP/MF/CC GO terms and KEGG pathways of DEPs between the capsules of different cultivars.

The DEPs obtained from comparisons of capsules among the cultivars were subjected to ontology and pathway analyses (Figure 5). Subcellular localization analysis revealed that most DEPs were in the cytoplasm. Gene ontology analysis indicated that these genes were significantly enriched for “translation” and “carbohydrate metabolic process” GO terms in BP. Specifically, it was found that protein transport and carbohydrate metabolism were more enhancedin Ofis_1 capsules compared to the control plant, whereas methylation and transport were more abundant in Ofis_NP than in the control plant (Supplementary Figure 3).

Similar to other KEGG analyses, the biosynthesis of secondary metabolites pathway was enriched more in the KEGG analysis (Figure 5). However, the carbon metabolism pathway was obtained only for DEPs observed between the capsules of noscapine-rich and morphine-rich cultivars.

### 3.3. Expression profiles of the known proteins in BIA pathway

Although the BIA pathway is highly complex, several genes have been identified in critical steps (Singh et al., 2019). The protein expression levels of these genes were also analyzed in the dataset, and the expected results were obtained. Specifically, expression levels of norcoclaurine synthase (NCS), 4′-O-methyltransferase (4′OMT) were found to be upregulated in stems compared to capsules, while Ofis_1 exhibited the highest expression among the cultivars. Salutaridine synthase (SalSyn) and neopinone isomerase (NISO) proteins, which function in the morphine biosynthesis branch, were predictably expressed at higher levels in morphine-rich Ofis_1 than in noscapine-rich Ofis_NP. Additionally, expression of scoulerine 9-O-methyltransferase (SOMT) was upregulated in Ofis_1 compared to in Ofis_NP. Salutaridiol 7- O -acetyltransferase (SalAT) showed higher expression in stems compared to capsules for all the cultivars. Thebaine 6-O-dimethylase (T6ODM) was more expressed in stems compared to capsules for Ofis_1 and Ofis_96. Similarly, 1,13-dihydroxy-N-methylcanadine 13-O-acetyltransferase (AT1), which functions in the noscapine branch, exhibited higher expression levels in Ofis_NP than in Ofis_1.

### 3.4. Protein-protein interaction analysis

The PPI networks for stems of cultivars were constructed and used to identify 3, 3, and 12 clusters of highly interconnected nodes in DEPs of Ofis_1/Ofis_96, Ofis_NP/Ofis_96, and Ofis_1/Ofis_NP comparisons, respectively (Supplementary Figures 4–6). The most significant biological processes and pathways were associated with metabolic pathways, biosynthesis of secondary metabolites, and carbon metabolism.

## 4. Discussion

Opium poppy is one of the oldest and most valuable medicinal plants. Various studies have been conducted to elucidate the biological processes related to the biosynthesis and accumulation of its alkaloids (Decker et al., 2000; Zulak et al., 2007; Zulak et al., 2009; Desgagne Penix et al., 2010; Onoyovwe et al., 2013). In the present study, we aimed to profile the proteins that are differentially expressed in the stems and capsules of three opium poppy cultivars with varying alkaloid content.

The proteome data revealed that the protein profiles of the stem and capsules are clearly separated. It is known that the biosynthesis of alkaloids occurs in sieve elements, while capsules are the major organ for the accumulation (Bird et al., 2003). Given the different functions of these organs, it is expected that various proteins participate in them, leading to alterations in their expression levels. This study represents the first report demonstrating differences in organ-based protein expression in mature poppy plants.

Functional enrichment analysis of the proteins upregulated in stems compared to those in capsules revealed a significant enrichment in photosynthesis-related proteins. Notably, the term “photosynthesis” was even higher when comparing the stem and capsule organs of varieties rich in alkaloids. Confirmation was obtained that the CC terms of the proteins were associated with the chloroplast thylakoid membrane, photosystem I, and photosystem II. It is known that light triggers the biosynthesis and accumulation of certain secondary metabolites (Kong et al., 2016; Thoma et al., 2020). It was shown that the application of a consortium of endophytes increased morphine and thebaine content, along with photosynthesis efficiency in poppy plants (Ray et al., 2019). In another study, it was reported that the application of Triacontanol led to increased morphine content and enhanced photosynthesis upon (Srivastava and Sharma, 1990). It has been proposed that poppy plants use a high level of photosynthetic assimilation and energy to synthesize more alkaloids (Kara, 2021). Our results, supported by the literature information, indicated that photosynthesis is the primary distinguishing biological process between stem and capsule organs in the poppy plant, and it is one of the pivotal processes in alkaloid biosynthesis.

The term “photosynthesis” was also found to be the most abundant term in the ontology analysis of stem proteins with increased expression in the Ofis_1 compared to the Ofis_NP. Following this, the second most enriched protein was associated with the “glycolytic process”. Furthermore, the results of the KEGG pathway analysis supported the findings of GO enrichment, indicating that carbon metabolism, an energy-related pathway, was enriched. These data suggest that the energy required for morphine biosynthesis is more significant than that needed for noscapine biosynthesis. One of transcriptomic data also supported our finding (Gurkok et al., 2015). It was shown that sucrose and starch metabolisms were significantly upregulated in the fungal elicitor-treated opium poppy. It is known that sucrose and starch are end-products of photosynthesis (Goldschmidt and Huber, 1992). Therefore, both transcriptomic and proteomic data strongly supported the influence of photosynthesis in BIA pathway.

Detailed analysis resulted that the protein whose expression increased the most in the stem of Ofis1 compared to both stems of OfisNP (9.6-fold change) and Office 96 (9.4-fold change) was the probable cinnamyl alcohol dehydrogenase 1 (CAD) protein (accession A0A4Y7K4R1). The CAD is involved in the reduction of cinnamaldehydes into cinnamyl alcohols, and members of CAD family serve as primary genes in lignin biosynthesis (Tronchet et al., 2010). It was shown that lignin deficiency resulted in a decrease in photosynthesis. When considering all this information, photosynthesis emerges as the pivotal process for BIA biosynthesis, specifically for morphine.

(S)-reticuline is the central intermediate in BIA metabolism. (S)- to (R)-reticuline conversion is a critical step in morphinan biosynthesis, and cytochrome P450 involves in this conversion (Winzer et al., 2015). Transcripts of cytochrome P450 were also found to be abundant in fungal elicitor-treated poppy cell cultures (Desgagne Penix et al., 2010). Cytochrome P450 enzymes constitute a family of heme-thiolate proteins, with several alkaloids being modified by these enzymes (Liu et al., 2020). Consequently, the heme binding function was found to be one of the most abundant MF ontology terms in our analysis.

The ontology analysis also revealed that the term “methylation”, which refers to the process in which a methyl group is covalently attached to a molecule, was abundant among the organs of alkaloid-rich cultivars. The involvement of O-methyltransferases in alkaloid biosynthesis is well-known (Dang and Facchini, 2012; Park et al., 2018; Agarwal et al., 2019). Supporting these studies, the abundance of the term methylation, especially in alkaloid-rich varieties, emphasizes the essential roles of the activity of O_methyltransferase in BIA biosynthesis in opium poppy. The increase in expression of 4′OMT enzyme in the stems of comparisons of Ofis_1 / Ofis_96 and Ofis_1 / Ofis_NP and the increased expression of SOMT enzyme in the stems of Ofis_1 and Ofis_NP compared to their capsules also strengthened those results. These results are consistent with the proteomic data, as indicated by one of the transcriptome studies showing that methionine metabolism was significantly affected in the elicitor-induced opium poppy. This observed effect correlated with the activity of methyltransferases, which have important functions in the BIA pathway (Gurkok et al., 2015). However, it should be noted that the “methylation” term also involves epigenetic regulations. Bulut et al. (2020) used the methylation-sensitive amplification polymorphism (MSAP) profiling technique to examine DNA methylation profiles among poppy varieties with different BIA contents. In that study, different methylation profiles in the capsule and stem organs of poppy cultivars showed that this epigenetic mechanism may be effective on BIA biosynthesis. Therefore, the possibility of epigenetic regulations on BIA pathway should not be overlooked.

Previous studies showed the inhibitory effect of noscapine on microtubule assembly in a colchicine-like manner (Nambiar et al., 2020; Oliva et al., 2020; Awasthi et al., 2022). It is known that translation machinery directly interacts with microtubules (Chudinova and Nadezhdina, 2018). Certain secondary metabolites have been identified as effectors of small molecule translation inhibition (Yamashita et al., 2017). Ribosomes serve as sensors for numerous metabolites, and specific metabolites usually promote ribosome stalling, and therefore metabolites can control translation (van der Horst et al., 2020). Our data indicated that proteins are downregulated in the noscapine-rich cultivar compared to both the morphine-rich and control cultivar. The proteins present in the stems, which exhibited an increase in the noscapine-rich cultivar compared to those in the stems of the other two cultivars, were found to be associated with translation. Supporting that, “ribosome” was the most enriched CC term for these proteins. Furthermore, a high proportion of Ofis_NP stem proteins, with increased expression relative to Ofis_1 stem, are involved in the structural constituent of the ribosome MF ontology term. Additionally, the stalling either provides time to fold or to be transported, or it may trigger the transport of degradation of proteins (Chyzynska et al., 2021). Thus, it is expected that transportation mechanisms are involved in this process. Supporting that, intracellular protein transport and vesicle-mediated transport were among the most significantly enriched terms for the upregulated stems proteins of Ofis_NP compared to Ofis_1. A previous study revealed that transcripts involved in transportation were abundant in elicitor-treated poppy cell cultures, leading to induced sanguinarine content (Desgagne Penix et al., 2010). It is known that sanguinarine is synthesized in the noscapine branch rather than the morphine branch; thus, the study further supports our hypothesis.

In our analysis, the polyadenylate-binding protein RBP45-like (PABP) (accession number A0A4Y7K114) protein exhibited the most increased protein expression in Ofis_NP stem compared to Ofis_1 stem, with a 7.3-fold change. Confirming our previous results, it was shown that PABP enhances translation termination (Ivanov et al., 2019).

We may hypothesize that some proteins are translationally downregulated in response to noscapine, and this inhibition effect of noscapine might be mediated through the ribosome. In opium poppy, the bifurcation in the biosynthetic pathway of morphine and noscapine has not been elucidated, and the restrictive impact of noscapine might be even involved in disparate branches of the BIA metabolic network.

## 5. Conclusion

In this study, the differences in the protein composition of the mature opium poppy cultivars with varying BIA contents isolated from capsules and stems were dissected for the first time. The screened DEPs in stems revealed that photosynthesis was seen as one of the key influential processes involved in BIA biosynthesis. Furthermore, the proteome profiles of the cultivars indicated that noscapine has a noteworthy effect on the translational process. The proteomics profiles generated in the study construct a database for future research on opium poppy and the BIA biosynthetic pathway.

## Supplementary Data

Supplementary Figure 1 Top five enriched BP/MF/CC GO terms and KEGG pathways of DEPs with increased expression in each cultivar in comparison between stem and capsule.

Supplementary Figure 2 Top five enriched BP/MF/CC GO terms and KEGG pathways of DEPs with increased expression in each cultivar in comparison between stems of different cultivar.

Supplementary Figure 3 Top five enriched BP/MF/CC GO terms and KEGG pathways of DEPs with increased expression in each cultivar in comparison between capsules of different cultivars.

Supplementary Figure 4 PPI network of Ofis_1 stem /Ofis_96 stem. Each color represented a cluster.

Supplementary Figure 5 PPI network of Ofis_NP stem /Ofis_96 stem. Each color represented a cluster.

Supplementary Figure 6 PPI network of Ofis_1 stem /Ofis_NP stem. Each color represented a cluster.
