# Supplementary figures and images for: Divergent proteomic profiles of opium poppy cultivars
Source: Turk J Biol. 2024 Feb 6;48(1):80–90. doi: 10.55730/1300-0152.2684 (PMC11042869; doi:10.55730/1300-0152.2684)

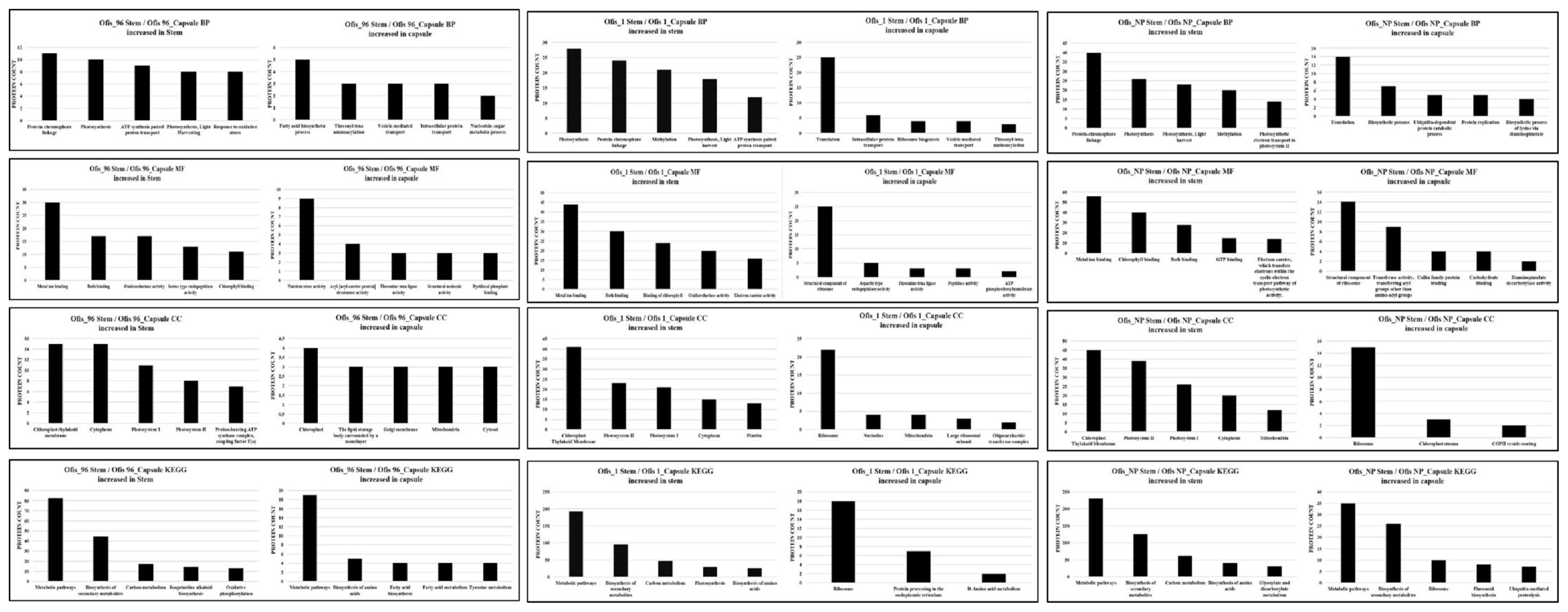

Supplement: Supplementary Figure 1 — Top five enriched BP/MF/CC GO terms and KEGG pathways of DEPs with increased expression in each cultivar in comparison between stem and capsule. [file tjb-48-01-080s1.tif]

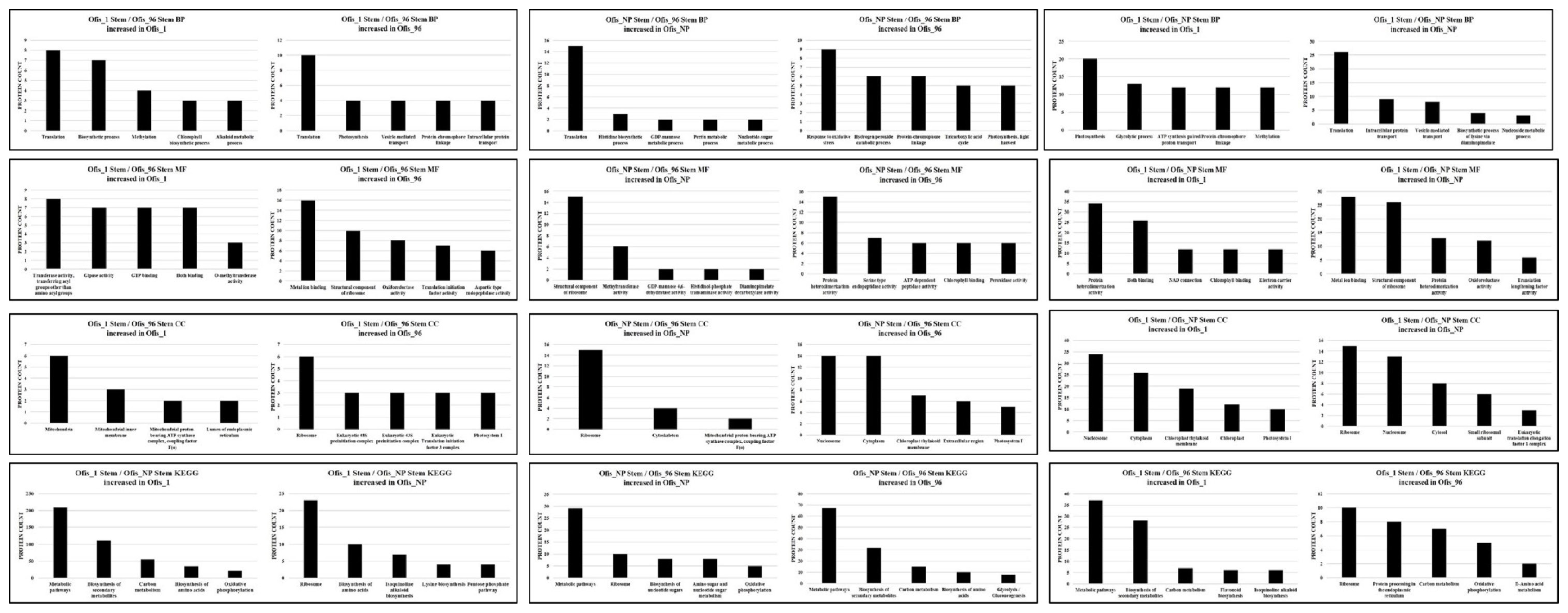

Supplement: Supplementary Figure 2 — Top five enriched BP/MF/CC GO terms and KEGG pathways of DEPs with increased expression in each cultivar in comparison between stems of different cultivar. [file tjb-48-01-080s2.tif]

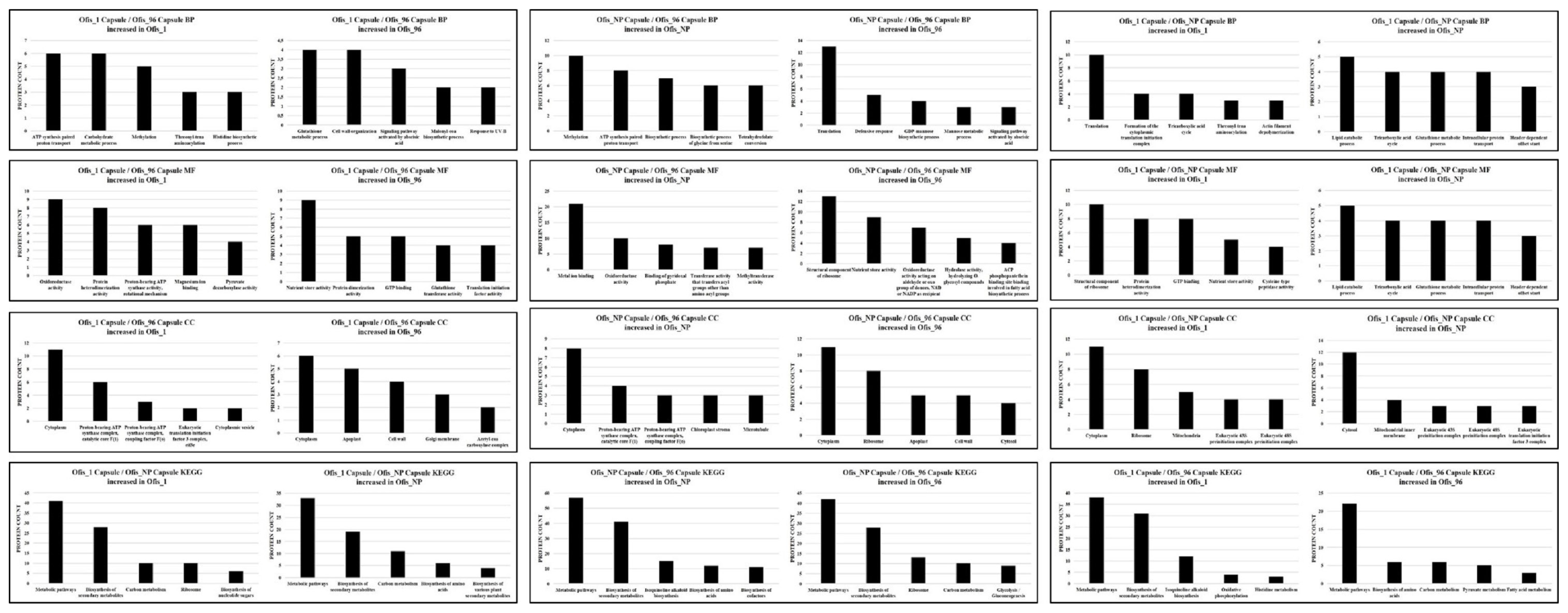

Supplement: Supplementary Figure 3 — Top five enriched BP/MF/CC GO terms and KEGG pathways of DEPs with increased expression in each cultivar in comparison between capsules of different cultivars. [file tjb-48-01-080s3.tif]

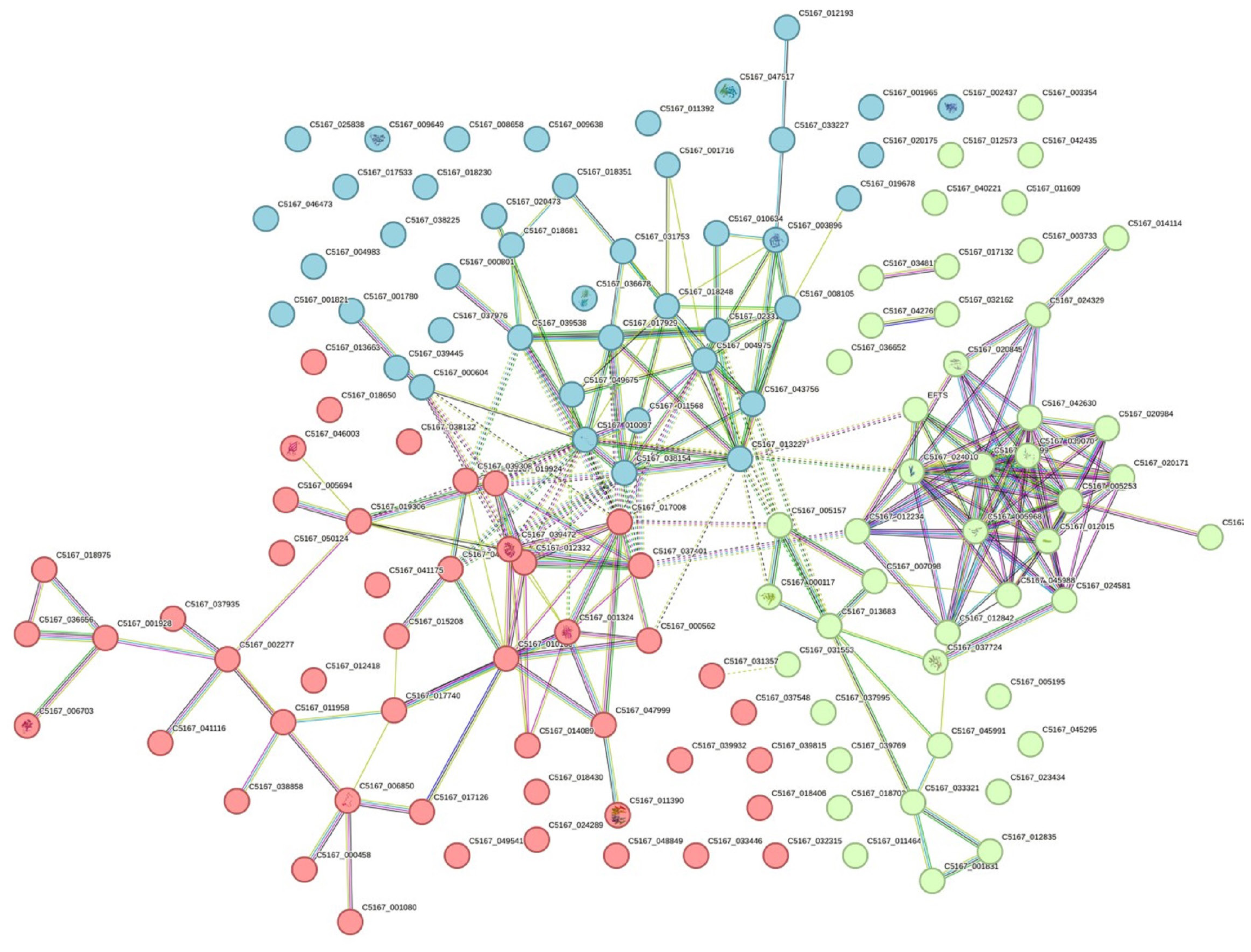

Supplement: Supplementary Figure 4 — PPI network of Ofis_1 stem /Ofis_96 stem. Each color represented a cluster. [file tjb-48-01-080s4.tif]

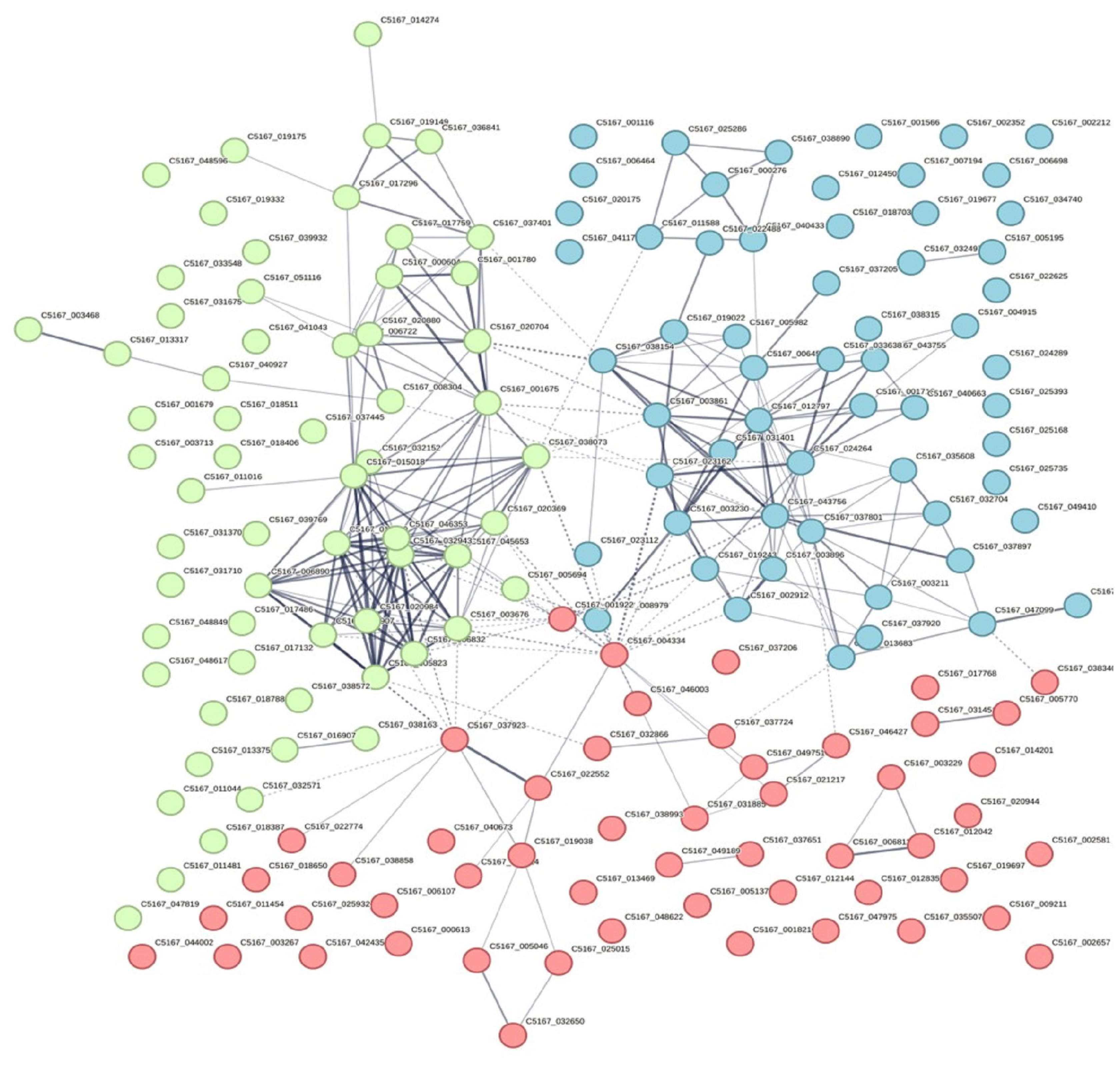

Supplement: Supplementary Figure 5 — PPI network of Ofis_NP stem /Ofis_96 stem. Each color represented a cluster. [file tjb-48-01-080s5.tif]

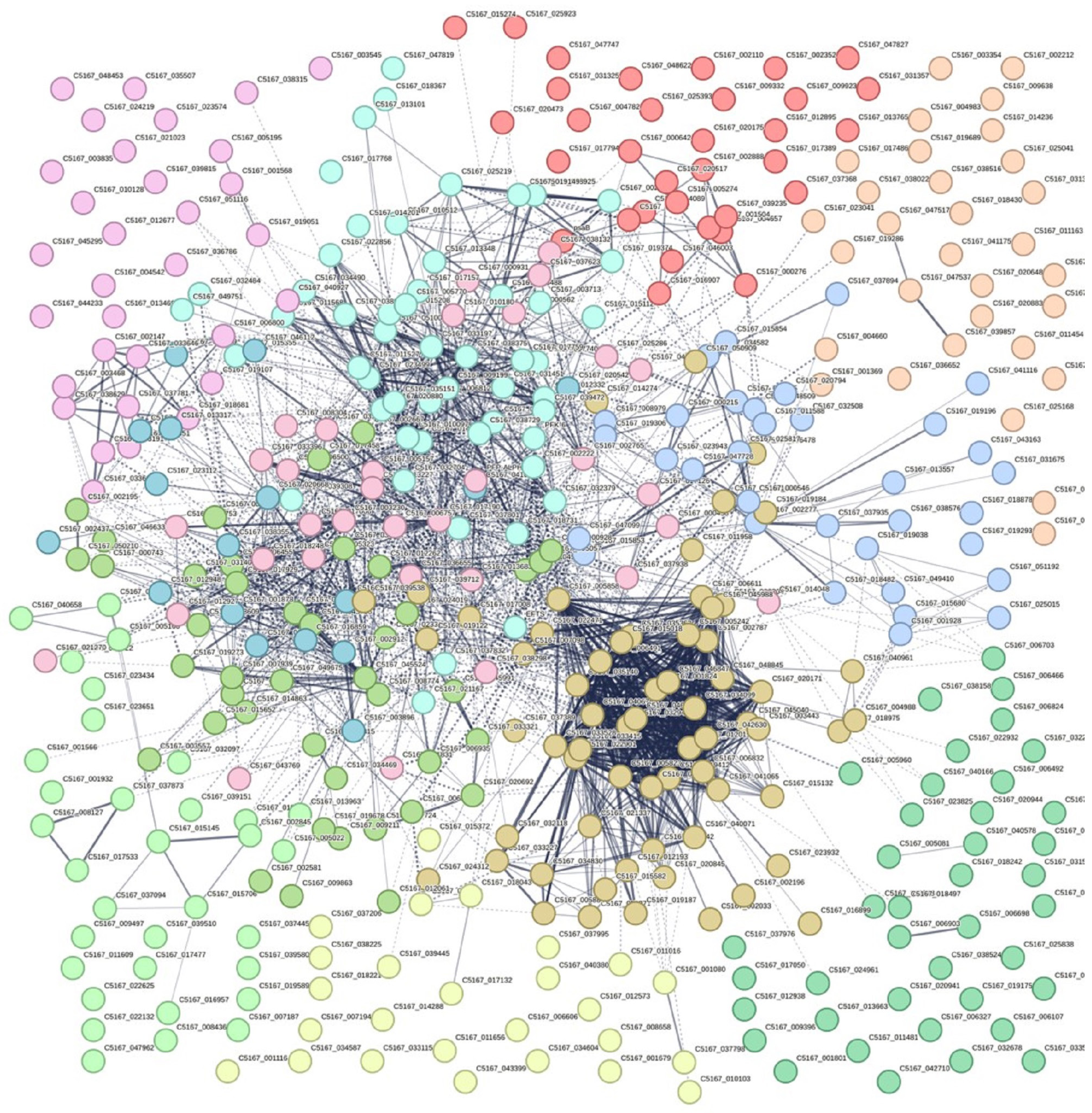

Supplement: Supplementary Figure 6 — PPI network of Ofis_1 stem /Ofis_NP stem. Each color represented a cluster. [file tjb-48-01-080s6.tif]
